# Mental health law: a comparison of compulsory hospital admission in Italy and the UK

**DOI:** 10.3389/fpubh.2023.1265046

**Published:** 2023-10-06

**Authors:** Lucienne Aguirre, Martina Padovano, Matteo Scopetti, Raffaele La Russa, Federico Manetti, Stefano D’Errico, Paola Frati, Vittorio Fineschi

**Affiliations:** ^1^City & Hackney Adult & Older People’s Mental Health Services, East London Foundation NHS Trust, London, United Kingdom; ^2^Department of Anatomical, Histological, Forensic and Orthopedic Sciences, Sapienza University of Rome, Rome, Italy; ^3^Department of Medical Surgical Sciences and Translational Medicine, Sapienza University of Rome, Rome, Italy; ^4^Department of Clinical and Experimental Medicine, University of Foggia, Foggia, Italy; ^5^Department of Medical, Surgical and Health Sciences, University of Trieste, Trieste, Italy

**Keywords:** compulsory admission, mental health law, mental health care, fundamental rights, public health

## Abstract

In Europe, the mental health law legal framework has had several changes throughout the years to achieve and develop new reforms, better mental health care, and protect the human rights of patients. The UK national data shows rising detention rates and the disproportionate use of the legal framework among people from black and minority ethnic groups. At the national level, compulsory admissions are lower in Italy; it also shows that it has increased in the last few years in both countries. The lack of ethnic national data, especially in Italy, limited the ability to understand compulsory admission, discrimination, and stigma in mental health. The present study aims to compare the legal framework of mental health law and compulsory hospital admission in Italy and the UK. A review of each country’s latest amendments to mental health law and the number of compulsory hospital admissions was conducted to understand the impact of changes in mental health care.

## 1. Introduction

Throughout the years, the European legal framework on compulsory admissions and mental health care has changed, with reforms and debates regarding the rights of a person with mental illness. According to the currently prevailing orientation, it would be desirable to adopt tools aimed at reducing stigmatization, developing international standards, producing guidelines, as well as raising awareness of the ethical challenges imposed by compulsory admissions.

Compulsory hospital admissions of patients with mental disorders are defined as admission to a psychiatric hospital without consent. England and Wales further consider the admission of an incapacitated person (1). The criteria for compulsory admissions can vary among different countries in Europe. The criteria can be the duration for compulsory admissions, the reason for admission (potential danger to self and others), and the need for treatment. For example, in countries such as Denmark, France, Portugal, and Spain, there is no maximum period for compulsory admission. Instead, in countries like Italy and the UK, there is a time limit for compulsory admission. For most European countries, the diagnosis of a mental disorder is necessary for admission (2). The second most common criterion is the need for treatment; Italy and Spain have implemented special legislation introducing the need-for-treatment criteria. The threat or actual danger to self or others is the most common additional criterion. Although previously Italian legislation focused on the legal obligation to protect society from the patient, it is interesting to note that Italy, and more recently Spain, removed the “danger to self and others” criterion from their reasons for compulsory admission (3). However, in countries like Denmark, Finland, Greece, Ireland, Portugal, and the UK, the consideration of the danger criterion (to self or others) is sufficient on its own (4). For example, refusing medication or treatment can be a sufficient reason to be admitted in countries like Belarus, Belgium, Finland, Ireland, Italy, and Russia. Finally, in Finland and Italy, a patient can be admitted if the outpatient procedure is not sufficient for the patient’s medical needs.

In such a context, the present study aims to compare the legal framework of compulsory admissions between Italy and the UK, reviewing national databases and the latest changes in legislation. For this purpose, a narrative literature on the updated epidemiological data and relevant national documents has been conducted.

## 2. Mental health law legal framework

Reports from around the world highlight the need to address discrimination and promote human rights in mental health services. For this purpose, careful discipline of coercive practices such as hospitalization and forced treatment, restraint (mechanical or pharmacological), or isolation is essential.

The United Nations Convention on the Rights of Persons with Disabilities (CRPD) marks a paradigm shift in the way disability and mental illness are conceptualized. It places the person at the center of the decisions that concern him and considers individuals as holders of rights based on equality with others. Such an approach to disability has profound implications for legal capacity legislation as well as its implementation. The CRPD recognizes the challenges outlined and calls for major reforms for the promotion of human rights through a fundamental paradigm shift in the field of mental health that includes the rethinking of rules, laws, systems, and services (5). Since the adoption of the CRPD in 2006, a growing number of countries are seeking to reform their laws to promote community inclusion, dignity, self-reliance, empowerment, and recovery. At present, however, few nations have established sufficient norms to implement the epochal changes required by the international model of human rights. In many cases, existing laws perpetuate a model based on institutionalization, isolation, and coercive therapeutic practices with patients exposed to poor quality care and human rights violations. To implement the changes required by the CRPD, it is essential to improve the integrated networks of mental health services in the community through drastic changes in the methods spread in many countries.

Approximately 50 million people with mental illness live in the European Union (EU). The restriction or deprivation of legal capacity to which many of these people are subjected constitutes an obstacle to the possibility of living independently and making free decisions about life. The CRPD has become an integral part of the legal order of the signatory countries, creating legal obligations regarding the prevention of discrimination and the protection of equality. Most existing laws on legal capacity share several common features; in fact, to limit the legal capacity of an individual, the presence of an intellectual disability or a mental health problem should be associated with a second criterion connected to the “impossibility” of the person to look after his own interests.

Despite many steps forward at the international level, the protection of mental health remains an issue rarely recognized as important. Safeguarding mental health deserves greater attention from society and states to guarantee individuals the best possible care. Achieving such a goal involves investing in policies and programs aimed at the active involvement of people with mental illness; in the same way, it is crucial to keep the concepts of mental health and physical health united to be able to proceed with an adequate allocation of resources.

### 2.1. Italy

In 1978 the Italian mental health care system underwent a radical change with the gradual closure of all the Mental Health Hospitals. The reform developed Departments of Mental Health nationally, delivering outpatient and inpatient care, running semi-residential and residential facilities, and having small psychiatric units in general hospitals.

Law 180 established four principles: (a) a gradual phasing out of mental hospitals; (b) the establishment of general hospital psychiatric wards for acute admissions, each having a maximum of 15 beds; (c) the restriction of compulsory admissions; and (d) the setting up of community mental health centers providing psychiatric care (6, 7).

The main principle of Law 180 is that patients with mental disorders have the right to be treated the same way as patients with other diseases, which means: (a) acute mental health conditions have to be managed in psychiatric wards located in general hospitals, (b) treatments should be provided voluntarily, and compulsory admissions kept only for specific circumstances, and (c) compulsory admissions need to be formally authorized by the Mayor and can only be undertaken in general hospital psychiatric wards (8).

To defend the need for treatment against a person’s will, Law 833/1978 (art. 33, 34, and 35) (9) stated that the need for compulsory admission must have an initial assessment by a medical practitioner and subsequently by a second physician belonging to the Local Health Unit. Both must confirm the presence of all the following criteria: (a) the patient shows mental changes requiring an urgent therapeutic intervention; (b) the patient does not accept the treatment; and (c) there are no conditions that provide adequate therapeutic measures outside those achievable in a hospital (10). Finally, the compulsory admission must be formally authorized by the Mayor of the municipality where the patient lives.

The Major has 48 h to complete the authorization and inform the municipality and patient of the admission. The compulsory hospital admission lasts for seven days, allowing the patient’s care, promoting voluntary treatment or less restrictive forms of compulsory intervention such as non-hospitalization (11). Compulsory admission could be theoretically renewed without definite time limits on a weekly basis if allowed by the Mayor and the Judge.

Despite the promulgation in 1978 of law 180 and of law 833 (“Institution of the National Health Service”), in Italy, there has been a lack of planning for years, especially in the field of organization of services. Such a shortage was partly due to the general difficulties associated with the implementation of the National Health Service.

Between 1994 and 1996 the reform process was reactivated, and the proposed intervention strategy provided a crucial framework to finally initiate a systematic reorganization of the services dedicated to psychiatric care. The main changes were (a) the establishment of the Department of Mental Health to guarantee unity and integration of psychiatric services within the same territory, (b) the identification of organizational components of the Department (territorial structures, hospital services, semi-residential facilities, and residential facilities), and (c) the activation of primary and secondary liaison care services. Due to ongoing uncertainties and delays by many regions, the Parliament, through repeated legislative interventions (financial laws of 1994, 1996, and 1997), introduced sanctions in the absence of operative interventions for the closure of psychiatric hospitals and the activation of mental health departments.

The Law 81/2014 outlined the closure of judicial psychiatric hospitals. The closure of judicial psychiatric hospitals, which ended in February 2017, involved their replacement with facilities called Residences for the Execution of Security Measures, with a limited number of beds in general hospitals.

### 2.2. England and Wales

The Mental Health Act 1983 (MHA) is the law in England and Wales under which a person with mental health problems can be admitted, detained, and treated in a hospital against their wishes (12). It tells what the people’s rights are regarding assessment and treatment in hospitals, treatment in the community, and civil or criminal pathways into hospitals. The MHA has been significantly amended since its introduction.

The Mental Capacity Act (MCA) was introduced in 2005 and was amended in 2007. The MCA is a legal framework that allows actions and decisions on behalf of vulnerable individuals who lack the mental capacity for self-determination (13). The Act states that it should be assumed that every adult (over 16 years old) has the capacity to make decisions for themselves unless this can be proven otherwise; this is also known as the presumption of capacity.

The Act set out five “statutory principles.” These values highlight the legal requirements in the Act (Table 1). The MHA also established the “Less Restriction” or the “least restrictive option” legal principle. Precisely, the MHA Code of Practice states: “*People taking action without a patient’s consent must attempt to keep to a minimum the restrictions they impose on the patient’s liberty, having regard to the purpose for which the restrictions are imposed*” (14). The code of conduct was reviewed in 2015 to encourage the least restrictive options of compulsory admission and the least duration.

**Table 1:** Comparison of the legal framework for compulsory admission between Italy and the UK.

	Italy	United Kingdom
Psychiatric hospitals	No	Yes
Psychiatric wards in general hospital	Yes	No
Community mental health team	Yes	Yes
Community mental health beds	Yes	No
Compulsory treatment regulation	Law no. 180/1978	Mental Health Act 1983 (amended in 2007)
Authorization	Initial assessment by two medical practitioners (one of whom is part of the Local Health Unit) The mayor can only formally authorize the admission under general hospital psychiatric wards and has 48 h to inform the patient	Two medical practitioners (one of whom with expertise in the management of mental disorders) and a Specially trained Approved Mental Health Professional (AMHP)
Duration of the hospital restriction	Up to 7 days Renewable only by the ward psychiatrist on a weekly basis	Admission for assessment (Section 2): up to 28 days Admission for treatment (Section 3): up to 6 months (renewable)
Involvement of a judge in reviewing the case after a certain period of time.	Yes	Yes

Therefore, before concluding that individuals lack the capacity to make a specific decision, it is important to take all possible steps to try to help them reach a decision themselves. Individuals have the right to make decisions that others might think are unwise. Action on behalf of an incapacitated person should be in the best interest and should be the option that least restricts fundamental rights.

In England and Wales, the circumstances in which individuals lawfully can be detained in a hospital for psychiatric assessment or treatment are defined in the MHA 1983 as amended 2007. Most compulsory psychiatric admissions are authorized on the grounds given in Sections 2 and 3 of the MHA. To be able to complete and authorize an MHA assessment two medical practitioners are needed (one of whom must be a psychiatrist) and a specially trained Approved Mental Health Professional (AMHP) who is a non-medical professional (often a social worker) and agrees on the application of legal criteria for compulsory admission.

The length of time an individual can be detained in the hospital depends on the mental health condition and the circumstances of each individual:

- Section 2 authorized the detention of a patient for up to 28 days. *“The patient suffering from mental disorder of a nature or degree which warrants the detention of the patient in a hospital for assessment for at least a limited period; and is in the interests of he/she own health or safety or with a view to the protection of other*” (12).
- Section 3 authorized the detention of a patient for up to 6 months and established the possibility of renewal. “*The patient should be suffering from mental disorder of a nature or degree which makes it appropriate for him/her to receive medical treatment in a hospital; and it is necessary for the health or safety of the patient or for the protection of other persons*” (12).

Once the regulatory principles have been outlined, it is easy to understand how operations cannot be separated from a careful evaluation of the clinical and legal criteria (Table 2).

**Table 2:** Statutory principles of the Mental Capacity Act 2005.

Five statutory principles of the Mental Capacity Act 2005	A person must be assumed to have capacity unless is established the lack. A person is not to be treated as unable to decide unless all practicable helpful steps have been taken without success. A person is not to be treated as unable to decide merely because he makes an unwise decision. An act performed or a decision taken under this law on behalf of an incapacitated person must be inspired by his best interest. Before any act or decision, the presence of alternatives that allow the achievement of the goal in a less restrictive way of the rights and freedom of action of the person must be evaluated.

The MHA in England and Wales has had several amendments since it was introduced in 1983. The Mental Health Amendment Act of 2007 proposed empowering healthcare professionals to detain, assess, and treat people with mental disorders in the interests of patient health and public safety; such a legal framework provides patients with guarantees on the appropriateness of treatments (15). In 2017 the MHA was reviewed due to concerns about rising detention rates, the disproportionate use of the Act among people from black and minority ethnic (BAME) groups, and some processes in the Act that were out of step with modern mental health system (16); it was finalised and published in December 2018. The Mental Health Bill 2022 has drafted a report with recommendations for further implementations that need to be addressed in the MHA. It states that the ability of patients to choose treatment is one of the most important measures to reduce detention and improve inequalities (17). The key reforms arising from the Bill are monitoring outcomes and cultural changes, advocating for patients and carers, speaking up about stigma, and finally, proposing to tackle inequalities in mental health services. Regarding the key changes to Section 2 (Admission for assessment) and Section 3 (Admission for treatment), it states:

- “*The new criterion requires an assessment that serious harm may be caused to the health and safety of the patient or others unless the patient is detained for assessment and treated*”.
- The replacement of the current definition of “appropriate treatment” with treatment that has “a reasonable prospect of alleviating, or preventing the worsening of, the disorder or one or more of its symptoms or manifestations”.
- The redefinition of “mental disorder,” which not constitute a reason for detention under Article 3 without a coexisting psychiatric illness.

The Bill has raised concerns about the latest data showing racial and ethnic inequalities in compulsory hospital admission. It reports that Black or “black British” people are four times more likely to be detained than people from “any white background.” The draft Bill was subject to pre-legislative scrutiny in December 2022 and published its report on 19 January 2023. The Committee supported the reform, and that the Government must strengthen the Bill to address rising detention rates and racial inequalities.

## 3. Epidemiological context

The implementation of the Italian reform determined a significant decline in compulsory admission from more than 20,000 in 1978 to less than 9,000 in 2015 (18). However, a different trend has been observed in some countries such as the United Kingdom where the implementation of the Mental Health Act resulted in a significant increase in compulsory admissions up to 58,400 in 2014–2015 (19).

According to the latest National Health System (20), there were 129,891 discharged adults with a diagnosis of mental disorder from Italian hospital facilities: 120,955 to primary care settings (93.1%) and 8,936 to community mental health teams (6.9%). Further. there were 5,538 compulsory treatments registered nationally in Mental Health Units, representing 7.0% of admissions to public psychiatric wards (78,950).

On the other hand, in the UK in 2020–21, 53,239 compulsory admissions were recorded, and 35,272 took place at hospital admission; additionally, 13,619 occurred following admission (20). It is estimated that the overall national totals will be higher with a likely increase in compulsory admissions of 4.5 percent from 2022.

In line with other studies and a national database, when comparing compulsory admission rates by gender, Italy and the UK show higher rates for males than females. As explained before, the latest National data in the UK reports that amongst the five broad ethnic groups, compulsory admission rates for the “Black or Black British” group (343.5 detentions per 100,000 population) were highest, compared to the White group. The “Any Other Ethnic Group” had the second highest rate of compulsory admission (502.2 admissions per 100,000 population) followed by the “Any Other Mixed Background” group (389.8 admissions per 100,000 population).

Unfortunately, there is a lack of ethnicity data in the national database regarding compulsory admission in Italy. Several studies in Italy have shown a higher rate of compulsory admission among migrants. A study conducted in a large metropolitan context demonstrated a prevalence of compulsory admissions in migrants more than double with respect to natives (23.24% versus 9.11%) (21). Another study showed that the risk of admission to a psychiatric unit is three times higher in migrants compared to non-migrants; compulsory admission among young migrants (15 to 24 years old) is four times higher. According to the same study, 20.7% of sub-Saharan Africans were admitted to psychiatric units versus 6.2% in the general population, and 17.9% of North Africans versus 8.7% in the general population (22).

## 4. Discussion

The principles of mental health law in different countries and the epidemiology of compulsory treatments raise concerns about patient care, treatment, and mental health services.

Interestingly in Italy, most of the information about ethnic groups and compulsory hospital admission is related to migrant studies and are not collected as part of a national database, which is more commonly seen in the British national database. The lack of ethnicity data, especially in Italy, limited our ability to understand the broader experiences of many minority communities regarding compulsory admissions. As studies have shown that compulsory admission rates are higher in migrants and minority ethnic groups, there are ongoing discussions about stigma towards mental health and ethnic inequalities in mental health services are ongoing.

Aside from ethical issues, the scientific evidence regarding compulsory treatment is currently heterogeneous. On the one hand, growing evidence demonstrates the long-term benefits of compulsory treatment with an improvement in symptoms, a reduction in suicidal ideation, relief from exacerbations of serious psychiatric illnesses and increased motivation to initiate treatment (23–25). On the other hand, compulsory treatment can damage the therapeutic alliance and reduce patient compliance with health care (26); weak therapeutic relationships can be extremely harmful for patients with psychiatric conditions. According to some authors, subjects undergoing compulsory treatment are at increased risk of coercive additional therapies and may experience anxiety or depression after discharge (27). Therefore, it is essential to conduct any compulsory treatment with a view to the safety and health of the patient. Precisely, the use of mandatory treatments must be oriented in a personalized way towards maximizing benefits and minimizing damages to protect the individual and the community (28). Although some studies highlight the clinical benefits of compulsory healthcare treatment, the lack of unambiguity of the evidence requires careful consideration of the expectations of improvement in individual cases given the compression of the fundamental rights of the individual.

Compulsory and coercive medical treatment profoundly affects the personal sphere of the individual, putting his integrity and dignity at risk. The absence of clear and uniform data makes this phenomenon difficult to analyze and monitor, particularly regarding the operating procedures carried out in the specific case. Compulsory hospitalization legislation must respect patients’ fundamental rights, reduce mental health stigma, increase awareness, and address discrimination and inequalities in mental health care. The legislative activity, therefore, must guarantee the utmost attention to the protection of personal freedom and health in the interest of the person and of the community (29–31). In essence, the personalization of compulsory treatments is fundamental to confine the limitations to the strict therapeutic necessity and favor the protection of rights. Likewise, a careful balance is essential for the dignity of the person and the principle of equality. Hence, further guidance and policies in mental health and capacity legislation must be considered to arrange a more robust legal framework for patient rights. In general, legislative efforts should tend not to segregate psychiatric legislation from public health, but to conceive it as an organic part of the regulation of compulsory treatments; the elimination of distinctions in the modalities and, in part, in the places of treatment is in fact fundamental in the affirmation of the principle of equality. Compulsory treatment must be considered an exceptional event, a derogation expressly authorized and regulated to the principle of the centrality of consent. Considering voluntariness to be fundamental and the obligation of treatment to be exceptional, every path concretely useful for reaching consent must be undertaken prior to compulsory treatment. The desire to create a broad framework of sanitary, administrative, and judicial guarantees must be based on the principle according to which coercive medical treatments must not violate the limits imposed by respect for the human person and his fundamental rights. Respect for the dignity of the person directly affects the methods of carrying out compulsory treatment, requiring strict criteria for assessing the behavior of health professionals. In codifying the modalities of compulsory treatments it is necessary to ask oneself whether the right to assistance is also identified with the recovery of the ability to develop one’s personality or whether it is in conflict with it; if a broad definition of health is accepted, as a state which permits an expansion of personality and intellectual resources, then the aim of treatment becomes that of recovering the capacities to exercise and enjoy the rights of the personality; in this way, the protection of the personality becomes the primary purpose of compulsory treatment, without being able to be limited anymore.

The improvement of the operating methods cannot be separated from comprehensive data collection and active surveillance of the procedures in place. The best approach to the subject must necessarily be based on a close relationship between science and law to obtain legislation that is as evidence-based as possible. To make the combination possible, the data must be of high quality, available (also through an adequate infrastructural and technological system), and monitored globally. In such an operational context, the role of scientific dissemination is crucial. The goal must be to guide the investments of time and money in political strategies oriented toward health decisions guided by the best scientific evidence available. So, monitoring the phenomenology of compulsory admission is desirable to recognize a chronological and socio-economic context; in other words, scientific activity is fundamental for orienting decisions in legal and health matters. From this perspective, the implementation of epidemiological data on compulsory hospitalization and BAME is of considerable importance to better understand social demographics and improve mental health care and patients’ rights. Healthcare professionals should by now be accustomed to maintaining an Evidence-Based approach in clinical practice. However, evidence-based medicine is not unanimously recognized and accepted as the best way forward in-patient care. Scientific publishers, journals, researchers, authors, science communicators, journalists, and communicators should share virtuous training and in-depth courses, defining methods and languages of scientific communication. Only through the sharing of intent will knowledge and the correct interpretation of scientific evidence be able to guide decisions in such a delicate matter. The attention and understanding of scientific evidence are essential so that political realities and decision-makers can adequately take charge of health policies. Concerning global social inequalities, particularly in terms of health determinants and health response to evidence, the incentive for systemic change is crucial. Frequently, the constant change in socio-economic and cultural contexts favors the simultaneous dissemination of qualitatively very different evidence, making the scientific dissemination of certainties and uncertainties very difficult. The insufficiency – if not even the complete absence – of strategies to prevent or counter misinformation on issues related to mental health and compulsory hospitalization requires the implementation of direct actions against misleading content. In light of the considerations made, not only the reliance on evidence-based medicine but also the convinced support of an evidence-based policy must be considered desirable.

Finally, it is important to consider further research, education, and multidisciplinary work aimed at continuously improving patient care and addressing racial and ethnic inequities for more culturally appropriate mental health services.

## Data availability statement

The original contributions presented in the study are included in the article/supplementary material, further inquiries can be directed to the corresponding author.

## Author contributions

LA: Conceptualization, Writing – original draft. MP: Conceptualization, Writing – original draft. MS: Conceptualization, Methodology, Writing – review & editing. RL: Methodology, Writing – review & editing. FM: Writing – original draft. SD’E: Methodology, Writing – review & editing. PF: Supervision, Writing – review & editing. VF: Supervision, Writing – review & editing.
